# TRIP Database 2.0: A Manually Curated Information Hub for Accessing TRP Channel Interaction Network

**DOI:** 10.1371/journal.pone.0047165

**Published:** 2012-10-11

**Authors:** Young-Cheul Shin, Soo-Yong Shin, Jung Nyeo Chun, Hyeon Sung Cho, Jin Muk Lim, Hong-Gee Kim, Insuk So, Dongseop Kwon, Ju-Hong Jeon

**Affiliations:** 1 Department of Physiology and Biomedical Sciences, Seoul National University College of Medicine, Seoul, Korea; 2 Department of Clinical Epidemiology and Biostatistics, Asan Medical Center, and University of Ulsan College of Medicine, Seoul, Korea; 3 Institute of Dermatological Science, Seoul National University, Seoul, Korea; 4 Interdisciplinary Program of Medical Informatics, Seoul National University College of Medicine, Seoul, Korea; 5 IT Convergence Technology Research Laboratory, Electronics and Telecommunications Research Institute, Daejeon, Korea; 6 Biomedical Knowledge Engineering Lab, Seoul National University, Seoul, Korea; 7 Department of Computer Engineering, Myongji University, Gyeonggi-do, Korea; Cinvestav-IPN, Mexico

## Abstract

Transient receptor potential (TRP) channels are a family of Ca^2+^-permeable cation channels that play a crucial role in biological and disease processes. To advance TRP channel research, we previously created the TRIP (*TR*ansient receptor potential channel-*I*nteracting *P*rotein) Database, a manually curated database that compiles scattered information on TRP channel protein-protein interactions (PPIs). However, the database needs to be improved for information accessibility and data utilization. Here, we present the TRIP Database 2.0 (http://www.trpchannel.org) in which many helpful, user-friendly web interfaces have been developed to facilitate knowledge acquisition and inspire new approaches to studying TRP channel functions: 1) the PPI information found in the supplementary data of referred articles was curated; 2) the PPI summary matrix enables users to intuitively grasp overall PPI information; 3) the search capability has been expanded to retrieve information from ‘PubMed’ and ‘PIE *the search*’ (a specialized search engine for PPI-related articles); and 4) the PPI data are available as sif files for network visualization and analysis using ‘Cytoscape’. Therefore, our TRIP Database 2.0 is an information hub that works toward advancing data-driven TRP channel research.

## Introduction

Transient receptor potential (TRP) channels are a superfamily of Ca^2+^-permeable cation channels that play a crucial role in a wide range of physiological processes [1], [2]. In mammals, the TRP channel family consists of 28 isotypes that are divided into 6 subfamilies based on structural and functional similarity [3], [4]: TRPC (canonical), TRPV (vanilloid), TRPM (melastatin), TRPP (polycystin), TRPML (mucolipin), and TRPA (ankyrin). TRP channels commonly translate various cellular stimuli into electrochemical signals, leading to changes in membrane potentials and intracellular Ca^2+^ levels [5].

Aberrant TRP channels have been implicated in various human diseases, such as genetic disorders, cardiovascular diseases, cancers, and neuropathic pain [6]–[8]. In addition, recent studies on mice with ablated TRP channels have provided insight into the causal role of TRP channels in the diseases [9], [10]. Therefore, TRP channels have attracted much attention as promising targets for therapeutic intervention in human diseases [11]. However, the molecular mechanisms through which TRP channels are involved in the disease pathologies are largely unknown.

TRP channel functions are regulated through interaction with many cellular proteins [12], [13]. Because pathophysiological phenotypes are determined by concerted protein-protein interactions (PPIs) [14], [15], accumulating yet scattered information on TRP channel PPIs should be gathered to assist in understanding the concise role of TRP channels in biological and disease processes. In this regard, we previously developed the TRIP (*TR*ansient receptor potential channel-*I*nteracting *P*rotein) Database, a manually curated database that aims to offer comprehensive information relevant to bench scientists on mammalian TRP channel PPIs [16]. However, the database left much to be desired in promoting access to information and stimulating the formulation of new hypotheses and experimental designs.

Since our database was initially published [16], we have endeavored to improve knowledge acquisition, expand data utilization, inspire a new way of thinking about TRP channel research, and adopt computational biology or bioinformatics tools. Here, we present the TRIP Database 2.0 (http://www.trpchannel.org), which serves as an information hub for access to the TRP channel interaction networks.

**Figure 1:**
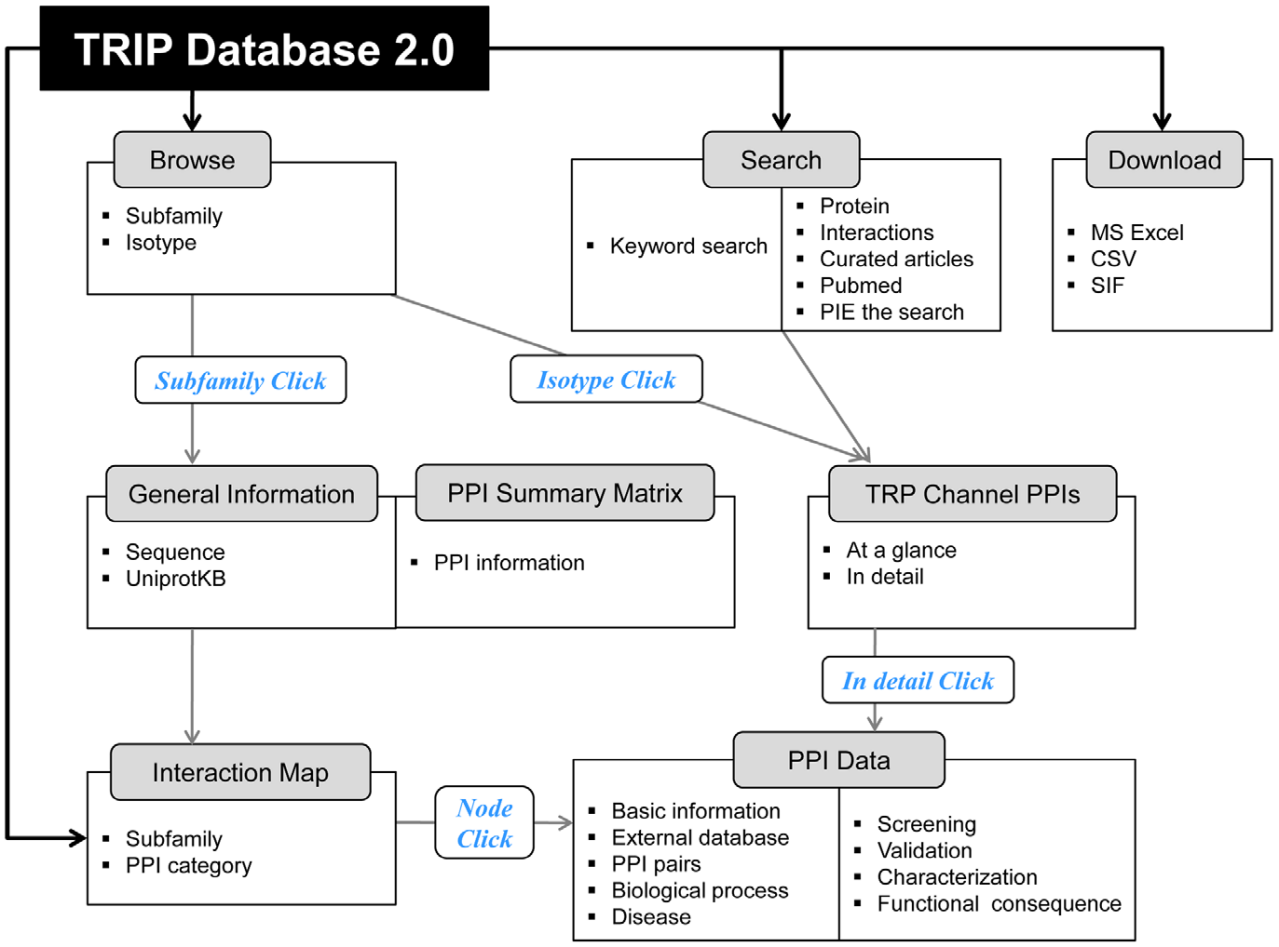
A diagram of navigating the TRIP Database 2.0. Users can browse the database based on TRP channel subfamilies or isotypes. If users choose to begin their search based on a specific TRP channel subfamily, they will get a summary of the subfamily, including general information (isotypes, amino acid sequences, or UniprotKB IDs) and PPI information, through the PPI summary matrix. If users choose to browse based on a specific TRP channel isotype, they will see lists of TRP channel PPI pairs. For example, the interface might provide one TRP channel with all of its interactors whereas in another instance all TRP channels with one interactor. Users also can start with a keyword search in the database or download the database contents in three formats.

## Design and Implementation

### Data Collection

TRP channel PPI data were manually collected through several steps as previously described (see the ‘IMPLEMENTATION’ section in our previous paper; [16]). Briefly, publications on TRP channel PPIs serve as the primary information source of the TRIP Database 2.0. We selected articles that contain experimental evidence for physical binding between the proteins indicated. All referred articles in the TRIP Database 2.0 are listed and can be accessed on our website (http://www.trpchannel.org/references). One of the most distinct features of the TRIP Database 2.0 is that the PPI data were collected from the supplementary data of the referred articles and the main data. In addition to a succinct summary of the PPI data, we collected in-depth information from the literature on relevant analytical methods and experimental resources for bench scientists.

**Figure 2:**
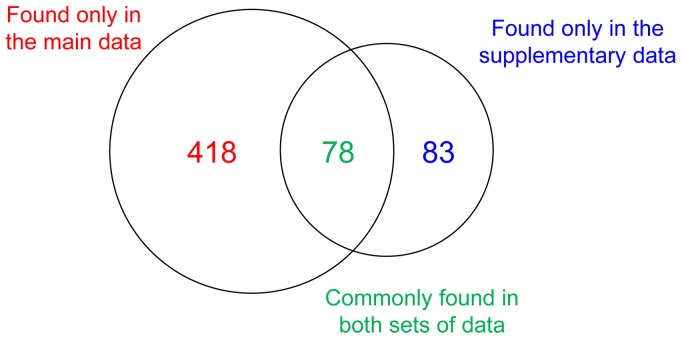
PPI information found in the supplementary data of referred articles. Of the total 579 PPI pairs listed in the TRIP Database 2.0, 83 PPI pairs were retrieved from only the supplementary data.

### Manual Curation

The PPI data are placed into 4 categories: screening, validation, characterization, and functional consequence (see the ‘CONTENTS AND DESIGN’ section in our previous paper; [16]). We also manually curated information relevant to bench scientists on analytical methods, experimental resources (e.g., genes, proteins, primary cells, tissues, and cell lines), and nomenclatures (see the ‘IMPLEMENTATION’ section in our previous paper; [16]). The TRIP Database 2.0 allows users to download our manual curation format as a Microsoft Excel file from our website (http://www.trpchannel.org/download).

**Figure 3:**
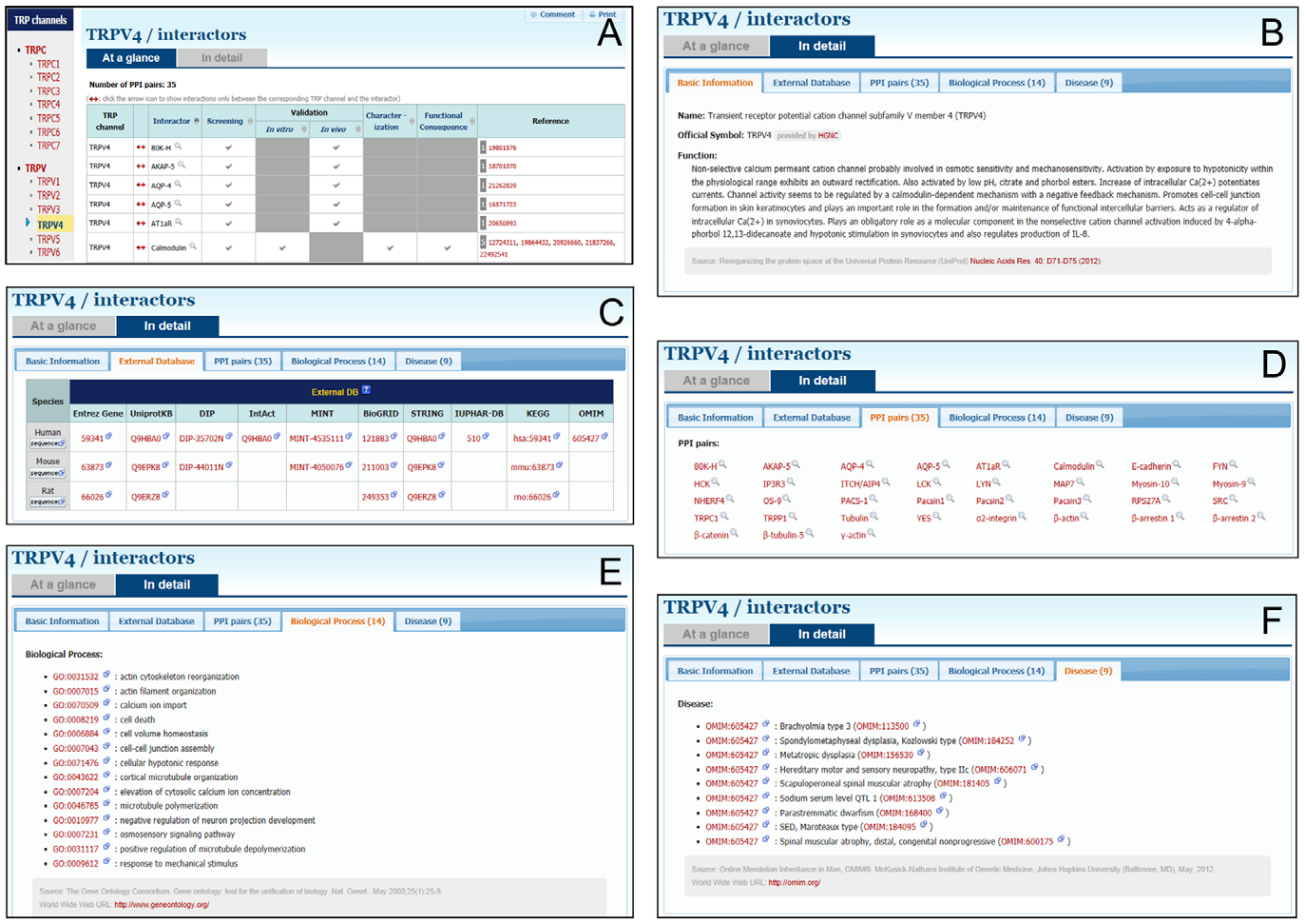
External links and data integration. Through clicking a specific TRP channel isotype (TRPV4 here) and then the ‘In detail’ button (A), users can see a tabular presentation, including basic information (B), external database (C), PPI pairs (D), biological process (E), and disease (F).

### Database Construction and Web Interface Development

The basic scheme of database construction and web interface implementation is the same as the previous version of our database [16]. Briefly, the data are stored on a MySQL server (version 5.1.63), an open-source relational database management system (RDBMS). The web interfaces were developed using Ruby on Rails 3.2.2, an open-source web framework, and JavaScript Libraries, such as jQuery 1.7.2 and jQuery UI 1.8.20. The web-based visualization tool was developed using Adobe Flex 4 SDK and Flare, an open-source data visualization library for Adobe Flash Player. BioRuby library [17] was used to access and integrate external data sources, such as UniprotKB (http://www.uniprot.org), HGNC (http://www.genenames.org), OMIM (http://www.omim.org), GO (http://www.geneontology.org), and PIE *the search* (http://www.ncbi.nlm.nih.gov/IRET/PIE). The web application runs on a Phusion Passenger 3.0.12 with an Apache HTTP server 2.2.14. The entire software system is hosted on an Ubuntu Linux server (version 10.0.4 LTS).

**Figure 4:**
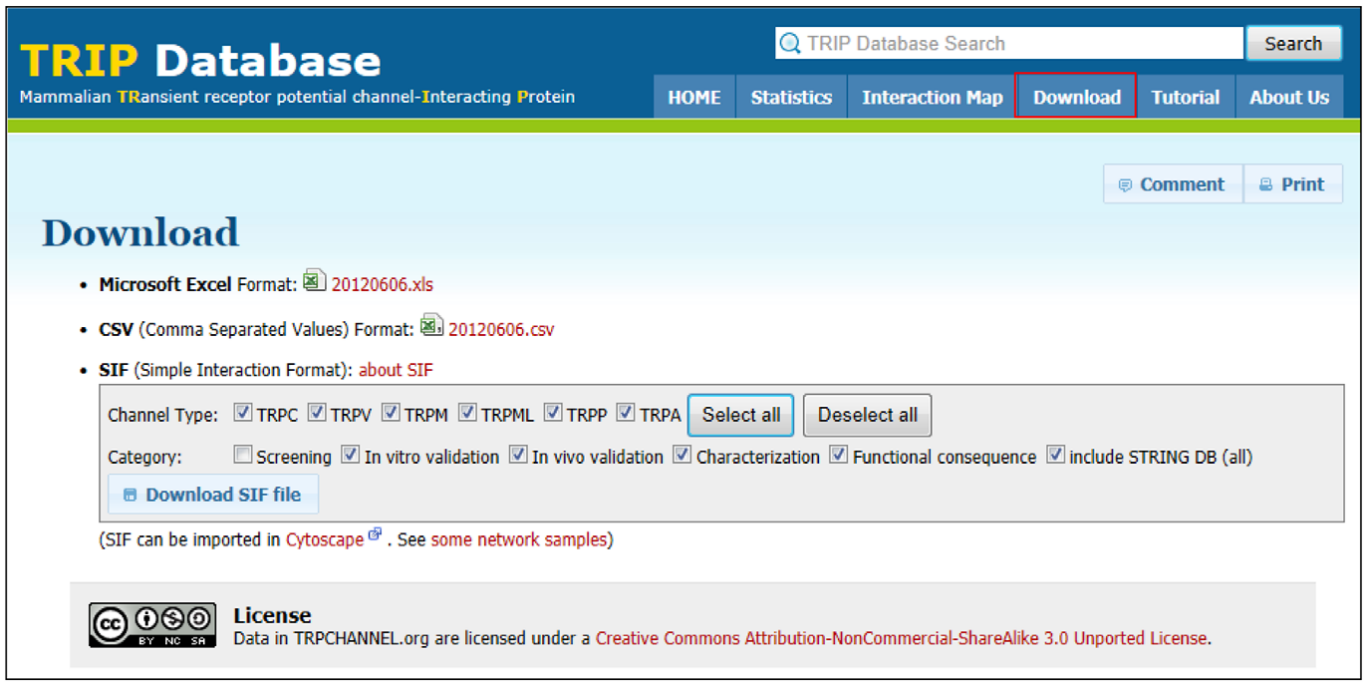
Downloading sif files. After clicking the ‘Download’ tab (red box), users can download various sif files according to option settings, such as TRP channel subfamily type and PPI category. In addition, our database offers the option to include the STRING DB contents for data enrichment. The TRIP Database 2.0 also provides a Microsoft Excel and a CSV file.

**Figure 5:**
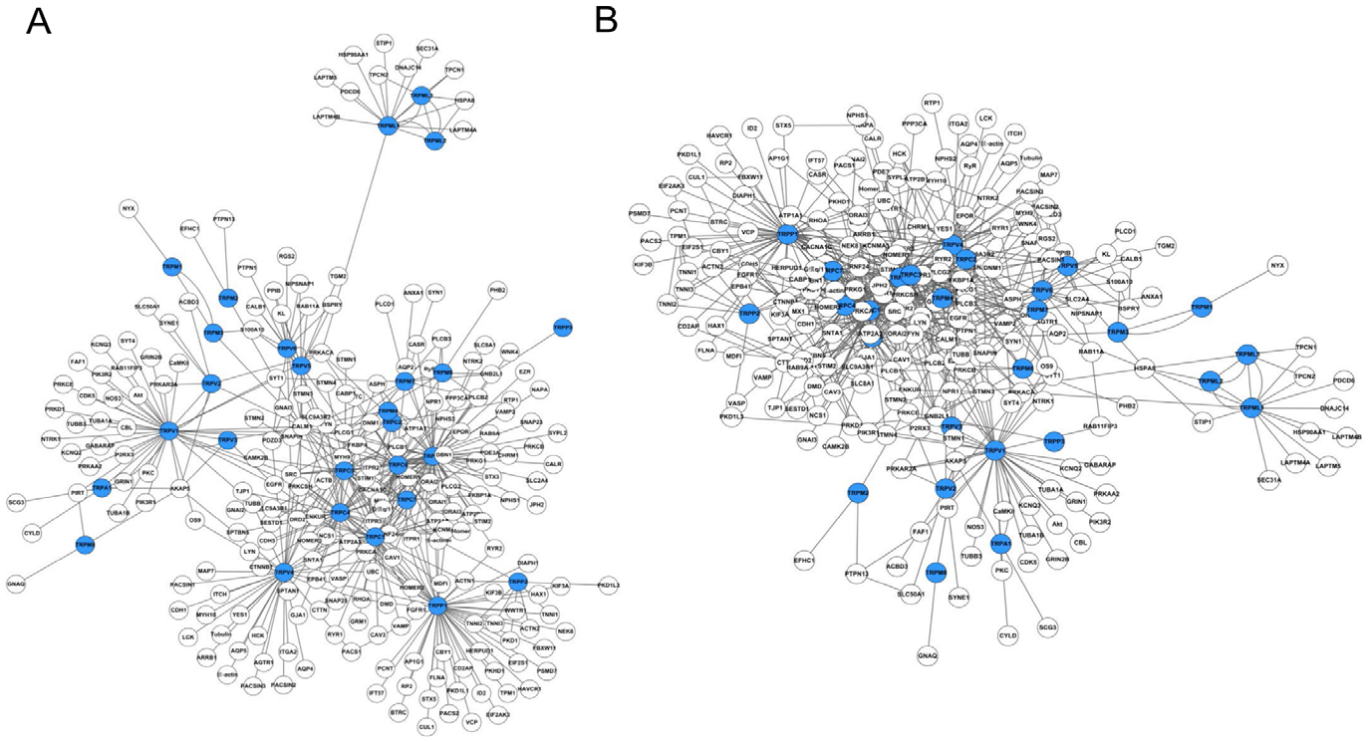
TRP channel PPI network. Users can represent the TRP channel PPI data as a network using the Cytoscape program. (A) The PPI network consists of the relations between TRP channels and their interacting proteins. (B) The extended PPI network includes two different relations: one between TRP channels and their interacting proteins and the other between the interacting proteins.

## Results

Examples of the TRIP Database 2.0 use are illustrated in Figure 1, and the website also provides instruction in the ‘Tutorial’ section (http://www.trpchannel.org/tutorial). The new distinct features of the TRIP Database 2.0 are described in detail below.

**Figure 6:**
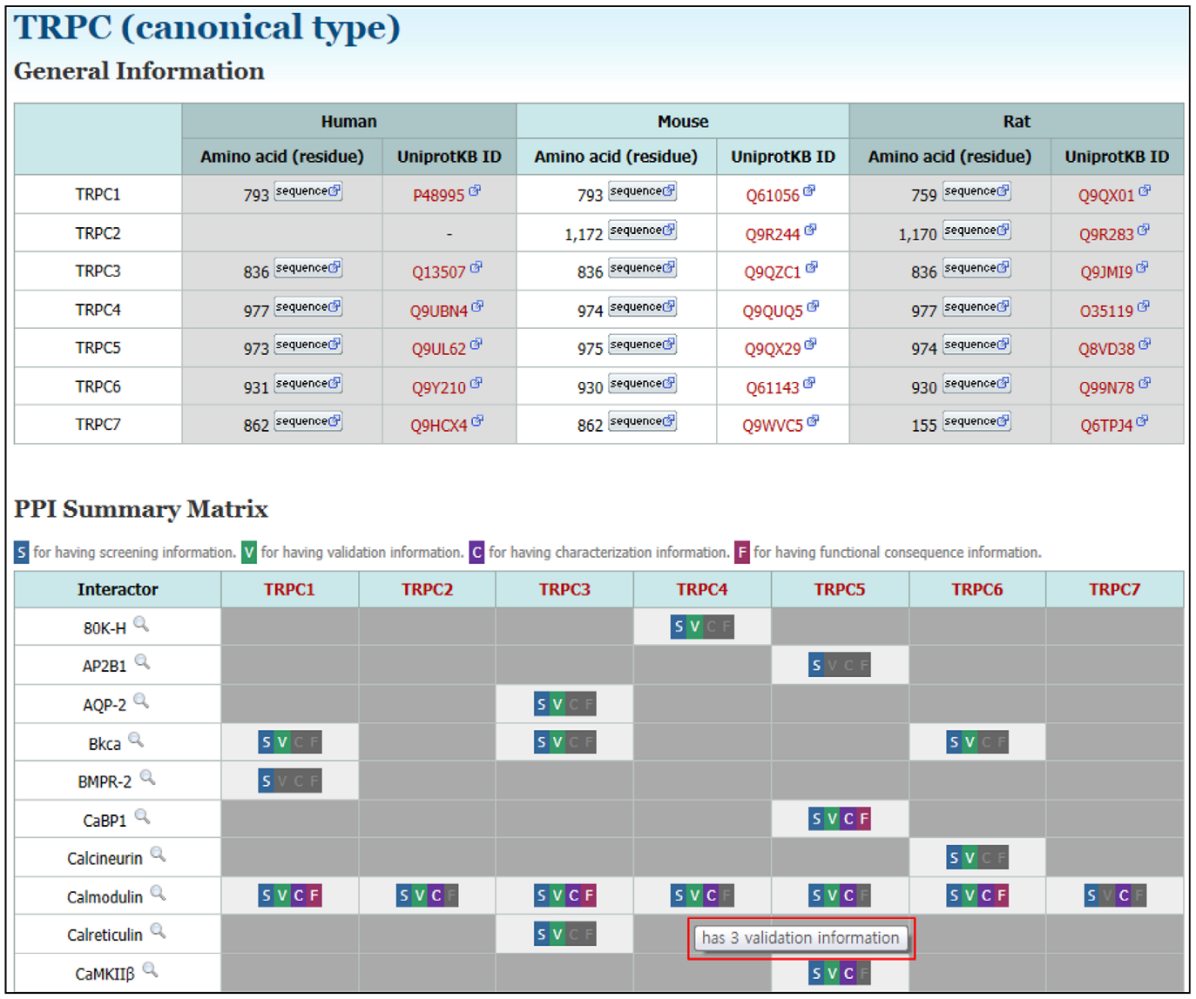
PPI summary matrix table. If users click TRP channel subfamily on the left panel, they can see ‘General Information’ and ‘PPI Summary Matrix’. General information provides UniprotKB IDs and amino acid sequences with FASTA, EMBL, or GenBank format. The PPI summary matrix enables users to intuitively grasp overall PPI information. Mouse pop-up help messages (red box) show the current state of information on data contents. By clicking each symbol (S, V, C, or F), users can obtain detailed information on each PPI pair.

### Database Contents

Since our first database was constructed [16], we have periodically updated its contents by manually curating new publications. Currently, the TRIP Database 2.0 has a 28% increase in the number of indexed articles (355 from 277 peer-reviewed publications), an 18% increase in the number of PPI pairs (579 from 490 PPI pairs), and a 15% increase in the number of cellular proteins (342 from 297 proteins). The documents included in the current version are also found in the ‘Statistics’ section (http://www.trpchannel.org/statistics). Users can download all PPI information in the TRIP Database 2.0 in a Microsoft Excel or CSV file with a Creative Commons License (http://www.trpchannel.org/download). These files provide a basic concept or framework for our manual curation schemes, which may assist in constructing similar biological databases.

**Figure 7:**
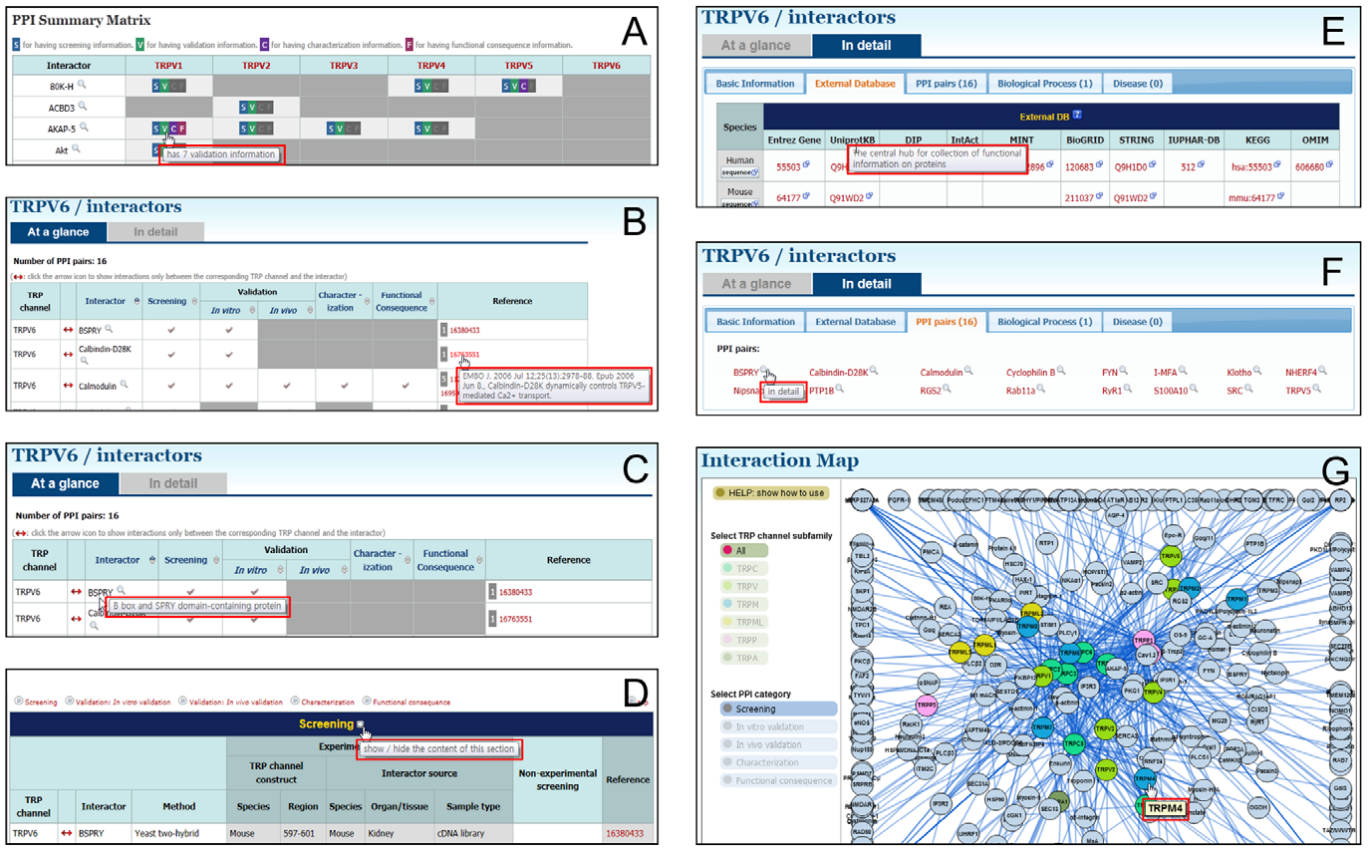
Mouse-over pop-up help messages. The TRIP Database 2.0 provides mouse-over pop-up messages, which are indicated as red boxes, to provide brief information of some icons. (A) Current state of information contents in the ‘PPI summary matrix’. (B) References in the ‘At a glance’. (C) Symbolic names in the ‘At a glance’. (D) Show/hide mark in the ‘In detail’. (E) External databases in the ‘In detail’. (F) PPI pairs in the ‘In detail’. (G) Node in the ‘Interaction Map’ pages.

The PPI data found in the supplementary data of the referred articles were scrutinized, collected, and curated in the TRIP Database 2.0. Of the total 579 PPI pairs listed in the TRIP Database 2.0, 418 PPI pairs were identified from only the main data, whereas 83 PPI pairs were retrieved from only the supplementary data of referred articles (Figure 2). A total of 78 PPI pairs were found in both sets of data. Because most current lab experimental articles include many supplementary data, particularly for high-throughput screen results (e.g., proteomic data), our efforts can provide an excellent example and a new direction for the manual curation of experimental research articles.

### External Links and Data Integration

To enable users to obtain additional and complementary information on each molecule, the TRIP Database 2.0 provides hyperlinks to useful external databases (see Tutorial 4 on our website). In addition, the database integrates basic key information on each molecule from external databases: the information on official symbols and functions for each molecule was retrieved from HGNC and UniprotKB and structurized, and the information on biological processes and diseases was extracted from GO and OMIM, and summarized. All of the contents described above are presented in a tabular format on our website (Figure 3). Taken together, the TRIP Database 2.0 serves as an information hub for access to functional, pharmacological, and pathophysiological information on the TRP channel interactome.

### Exporting the TRIP Database 2.0 Contents with Bioinformatics Tools

Network-based approaches uncover the organizing principles that govern biological systems and offer a new conceptual framework for understanding disease pathologies or discovering therapeutic strategies [14], [18], [19]. However, TRP channel network biology is still in its early stage. To promote future research on TRP channels, database contents should be easily available to well-known computational biology or bioinformatics tools. To this end, we offer our PPI data in sif files for network visualization and analysis using ‘Cytoscape (http://www.cytoscape.org)’, a popular bioinformatics package for visualizing biological network and integrating biological data [20]. If users are not familiar with Cytoscape, they can also view the TRP channel PPI network in the ‘Interaction Map’ section (http://www.trpchannel.org/visualization; the ‘HELP’ menu on the upper left side of the toolbar explains how to use the Interaction Map).

Users can download various sif files in the ‘Download’ section (http://www.trpchannel.org/download) (Figure 4). In particular, we offer an option to include the PPI information found in the STRING database (http://string-db.org) for data enrichment. This enables users to integrate PPI information among TRP channel interactors to expand the TRP channel PPI network. As shown in Figure 5, users can obtain TRP channel PPI network from the downloaded sif files using Cytoscape. Some network samples are also shown on our website (http://www.trpchannel.org/network_samples). Looking forward, we will continue to support other bioinformatics tools based on users’ requests and our internal needs to assist in stimulating network-guided hypothesis formulation and experimental designs.

### PPI Summary Matrix Table

To improve information accessibility and readability, we created a PPI summary matrix table in the TRIP Database 2.0. As shown in Figure 6, the columns and rows in the table are labeled with TRP channel isotypes and their interactors, respectively. Each corresponding box contains icons that represent the category of PPI information: screening (S), validation (V), characterization (C), and functional consequence (F). By clicking each symbol (S, V, C, or F), users can view the detailed information of each PPI pair. With the PPI summary matrix table, beginners in TRP channel study can instinctively grasp the current state of TRP channel PPIs. Taken together, the PPI summary matrix table is an excellent representative example for improving information accessibility and readability.

### Search System

The TRIP Database 2.0 provides search capability for proteins, PPIs, and relevant articles as previously described (see the ‘WEB INTERFACES’ section in our previous paper; [16] and Tutorial 5 on our website). In the TRIP Database 2.0, the search capability has been expanded to retrieve information from ‘PubMed’ and ‘PIE *the search*’, a specialized search engine for PPI-related articles [21]. Therefore, for given search words, the TRIP Database 2.0 presents corresponding information retrieved from external sources on the fly.

### Other Helpful Features

Based on users’ requests, we have implemented mouse-over pop-up messages to provide brief information on some icons in the tables and references (Figure 7). To encourage interactive communication with users, we have also added a ‘Comment’ menu on each page. Now, users can make comments on entries in the TRIP Database 2.0. We hope that these features will greatly improve the reliability of the database contents, and the participation of researchers with diverse specialties will accelerate interdisciplinary research, discover new principles and create new values in TRP channel research.

## Discussion

In this paper, we described the major new features of the TRIP Database 2.0. The TRIP Database 2.0 can serve as an information hub site for providing views on the molecular landscape of TRP channel PPI network, and assist those seeking to gain insight into the molecular mechanisms through which TRP channels are associated with a variety of biological and disease processes. Our Excel and CSV files will aid researchers in summarizing and representing TRP channel PPI data. The PPI summary matrix table is an excellent example for improving information accessibility and readability. Furthermore, our sif files will facilitate data-driven research for novel biological discoveries. We have no doubt that TRIP Database 2.0 will play a key role in paving a new way of investigation of TRP channels, and it can be used as a useful tool by TRP channel biologists and computation researchers.

There are several much needed upgrades for TRIP Database 2.0 other than periodic content updates: 1) the increased interoperability of database contents by adopting Proteomics Standards Initiative Common QUery InterfaCe (PSICQUIC), a standard web service to access and query molecular interaction databases programmatically [22]; a more formal representation mechanism, such as ontology, to represent TRP channel PPIs; 3) the development of new web-based programs, such as network analysis tools based on the network theory; and 4) the investigation and discovery of new roles of TRP channels in biological disease processes by integrating other biological databases, such as metabolic and microRNA databases, in addition to other global PPI databases.
